# Deep Convolutional and LSTM Networks on Multi-Channel Time Series Data for Gait Phase Recognition

**DOI:** 10.3390/s21030789

**Published:** 2021-01-25

**Authors:** David Kreuzer, Michael Munz

**Affiliations:** Institute for Medical Engineering and Mechatronic, Ulm University of Applied Sciences, 89081 Ulm, Germany; david.kreuzer@thu.de

**Keywords:** gait analysis, deep learning, LSTM networks, convolutional neural networks, ConvLSTM networks, gait phase detection

## Abstract

With an ageing society comes the increased prevalence of gait disorders. The restriction of mobility leads to a considerable reduction in the quality of life, because associated falls increase morbidity and mortality. Consideration of gait analysis data often alters surgical recommendations. For that reason, the early and systematic diagnostic treatment of gait disorders can spare a lot of suffering. As modern gait analysis systems are, in most cases, still very costly, many patients are not privileged enough to have access to comparable therapies. Low-cost systems such as inertial measurement units (IMUs) still pose major challenges, but offer possibilities for automatic real-time motion analysis. In this paper, we present a new approach to reliably detect human gait phases, using IMUs and machine learning methods. This approach should form the foundation of a new medical device to be used for gait analysis. A model is presented combining deep 2D-convolutional and LSTM networks to perform a classification task; it predicts the current gait phase with an accuracy of over 92% on an unseen subject, differentiating between five different phases. In the course of the paper, different approaches to optimize the performance of the model are presented and evaluated.

## 1. Introduction

Even though walking is the most basic method of locomotion, independence and self-determination are linked to it. Abnormal gait patterns, often caused by occasional pain, injuries or surgeries in the past, can lead to serious damage in the lower limbs and back [1,2,3]. Early detection of wrong movements can help to prevent worse conditions. Instead of fighting the symptoms, it can be more helpful to identify the underlying cause by analyzing the movement patterns. These patterns can be found and evaluated with the use of gait analysis methods. Nowadays, gait analysis is primarily performed by visual experts’ valuation in real time or by using specialized measurement equipment. The use of supporting systems offers the advantage of more accurate and data-driven evaluations. Systems such as SIMI-motion (Simi Reality Motion Systems GmbH, Unterschleißheim, Germany), Qualisys Track Manager (Qualisys AB, Göteborg, Sweden) abd Zebris FDM-T system (zebris Medical GmbH, Isny, Germany) are often used in modern gait analysis laboratories. They rely on either marker-based video capturing systems or foot pressure-based measurement systems, which combine a pressure plate with a treadmill. The downsides of these systems are that they can only be used in a stationary way, are limited in time, are expensive and require costly post-processing steps, making online usage difficult. This problem can be solved by the usage of inertial measurement units (IMUs).

Wearable sensors have become increasingly popular in recent years [4]. Smart devices, such as smartwatches, smartphones and smart glasses, already gather a tremendous amount of data on human movement by using the IMUs, which can integrate a 3D-accelerometer, a 3D-gyroscope, a magnetometer, a barometric pressure sensor and sometimes even electromyography (EMG) sensors. This provides the possibility to make long-term measurements with small, cost-efficient devices. Nevertheless, until today wearable sensor units have rarely been used when analyzing human gait in a clinical environment due to several reasons. The first difficulty is to automatically divide the continuous signal into the existing gait phases before an evaluation of further parameters is possible. To solve this problem, Hidden markov models (HMMs) [5,6] or rule-based approaches are commonly used, oftentimes only detecting the heel strike (HS) and toe off (TO) and discarding the extra information, such as the division of inner phases [7]. In 2014, a rule-based algorithm reaching over 90% accuracy tackling this task was presented by Goršič et al. [8]. As the human gait is highly individual, conventional algorithms often fail to generalize when depending on threshold values, as can be seen in this case. Thresholds have to be adapted to each user individually. To solve this problem, deep learning, which denotes the use of multi-layer neural network models, is often used. Using deep learning for activity recognition and gait analysis is a very active field of research. The main focus of the field lies in handling time series data. For time series data in general, long short-term memory (LSTM) networks are often used because of their ability to carry past information for current predictions [9]. In 2020, Vaith et al. [10] published an approach to predict single and double support phases using an LSTM network in IMU input data and active learning methods. Other approaches are based on convolutional neural networks (CNNs), using time segments as input. These segments are generated by defining a window with a fixed size and sliding it across the time series [11,12]. In this paper we combine convolutional and LSTM networks (ConvLSTM). In [13], a common activity recognition dataset was analyzed with a ConvLSTM model and compared to the baseline CNN model. It can be seen that the ConvLSTM outperforms the baseline CNN model in terms of F1 score performance for all chosen tasks. In [14], again, a ConvLSTM was used for human identification with IMU sensors, showing that this type of network shows a good performance in processing IMU sensor data. As the data were recorded under unconstrained conditions, steps had to be segmented first. This was done without the use of machine learning methods. Gait phases were not separated. In 2018 an approach to detect gait phases with IMU sensors was published by Martinez-Hernandez et al. [15]. They gathered data of 12 subjects, separating each step into eight periods. To detect the beginning and end of a step, two foot pressure-insole sensors were used. With this setup, an accuracy of 98.32% was reached. Unfortunately no sensory ground truth was used to detect the inner gait phases, leading to gait phases between HS and TO being assumed to be equidistant, making the calculation by far simpler, as the complexity of this setup is not significantly different from detecting initial contact, since equidistant inner periods can be separated manually.

In 2020 another approach using a CNN was presented by Jeon et al. [16] reaching 99.8% accuracy, but having a huge drop in accuracy when testing with unseen data. In this paper, a new ConvLSTM approach is presented to automatically divide the time series into its gait phases, with higher granularity and individuality compared to other papers before. This then serves as a basis for later automatic or manual gait analysis, with the claim that the temporal assignment of a regarded signal to the gait cycle is more accurate and stable than approached in other publications. Since errors can have particularly serious consequences in medical applications, the output and possible sources of error are analyzed and the model is optimized to minimize mispredictions. In addition, the certainty coming along with the predictions is taken into consideration as an additional feature by ignoring uncertain predictions. Furthermore, we try to reduce the number of parameters of the model, by visualizing the feature importance during prediction and eliminating unused features. This should allow faster calculation to enable the possibility for online usage. The paper is structured as follows: In Section 2 the methods are presented, involving the sensor setup that has been used, the label generation process, the network architecture and the training routine. Section 3 shows how the trained network performs on new data. Here the results are analyzed with focus on the questions of why mispredictions occur, which features are most important and how the risk coming along with mispredictions can be minimized. In Section 4 the results are discussed, and a conclusion is drawn in Section 5.

## 2. Materials and Methods

### 2.1. Inertial Measurement Units and Sensor Setup

For this paper all the data were recorded at the THU (Ulm University of Applied Science, Ulm, Germany). Since predictions were made based on IMUs, all measurements were with the Shimmer3 Consensys IMUs (Shimmer Research Ltd, Dublin, Ireland). These consist of an accelerometer (±8.0 g), a gyroscope ($\pm 250\;\frac{{}^{\circ }}{sec}$), a magnetometer and a barometric pressure sensor. A sampling rate of 120 Hz was used for all measurements. The measurements were made using 11 IMUs. The IMUs were attached to the foot, tibia, thigh, hip and arm on each side of the body. One sensor, the master, was only used for synchronization and functioned as a reference for the current air pressure. The slaves were synchronized via Bluetooth with the master, which also received a 3 V trigger edge signal. Master/slave architectures are commonly used to ensure synchronicity across sensor units [17]. The treadmill and master IMU were triggered simultaneously to ensure synchronicity across the systems. For classification, 7 channels were used: acceleration and rotation in x, y and z directions, and barometric pressure data. In order to get an adequate amount of data, 11 subjects were recorded, being between 20 and 34 years old and 160 and 203 cm tall. The subjects consisted of 3 women and 8 men. All subjects were healthy and did not have any relevant physical restrictions.

### 2.2. Label Generation

**Figure 1:**
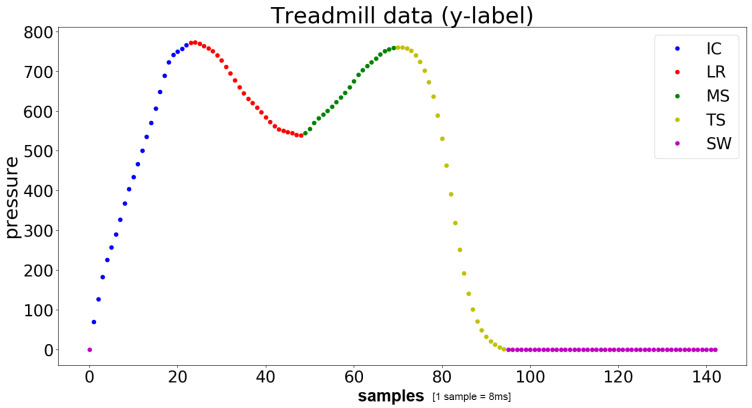
The pressure curve of one step, measured by the treadmill. The different colors indicate the gait phases used in this work. On the x-axis the time is given in frames (1 frame = 8 ms). The y-axis indicates the absolute pressure accumulated on the plate.

To record and calculate the labels for the IMU training data, a hpcosmos treadmill (mercury) was used, in combination with an FDM-T pressure plate attached below the treadmill belt, manufactured by Zebris (Zebris Medical GmbH). An alternative to using a pressure plate sensor would be a video-based motion analysis system based on markers. Although this technique could improve the accuracy, the pressure plate measurements are valid for generating the labels according to gait phases for this application. In contrast to this, video-based systems normally lead to a large overhead of applying markers, cross-calibration of cameras and manual post-processing steps. The software provided by Zebris allowed the extraction of the pressure distribution on the plate and some preprocessed parameters, such as the global step length and step width. The overall measured pressure allows for inference of the gait phases. Naturally, the pressure data do not provide further information about the swing phase and its sub phases, as the pressure was zero during the whole swing phase. The label of the corresponding class was assigned to every timestamp. Local maxima and minima help with finding the defined gait phases; see Figure 1. The signal is split into five phases: initial contact to loading response (IC), loading response (LR), mid stance (MS), terminal stance (TS) and swing phase (SW). The signal is synchronized with the IMU signal, by receiving a simultaneous trigger signal, which starts the pressure plate measurement. This start trigger signal is received in the analog–digital converter channel of one IMU sampled at 120 Hz, which serves as a synchronization trigger. This leads to a maximum time uncertainty of 8 ms regarding the trigger signal. An additional time uncertainty of one time frame of the IMUs (8 ms) has to be expected in the result. Synchronization between all IMUs is done using the synchronization approach developed by Shimmer. This approach is based on the estimation of clock offsets between the sensors and a master sensor by consecutively exchanging timestamps via Bluetooth. As the measurements were comparatively short, long-term errors in the on-board oscillators (drift) of the sensors did not need to be taken into account.

Considering the initial contact, a gait phase might not seem intuitive, since it denotes a single gait event. Stretching it to more than one timestamp was done to prevent the dataset from being unbalanced. The first timestamp in the defined IC phase, which is the actual event, can then be easily determined.

### 2.3. Deep Neural Network Architecture

**Figure 2:**
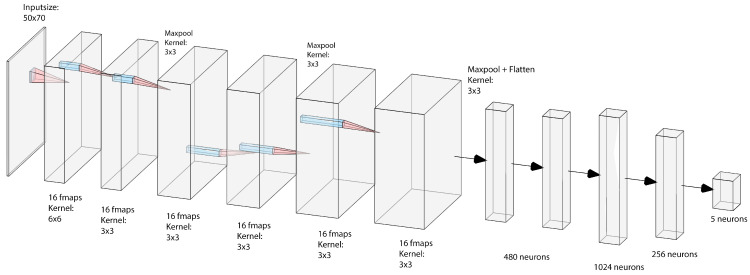
The structure of the neural network. Features generated by 6 convolutional layers are flatted and serve as input to 2 LSTM layers and 3 dense layers, including the output layer.

As described above, a combination of a convolutional neural network (CNN) and LSTM was used as a baseline model. An overview over the network architecture is shown in Figure 2. For the sake of brevity, we refer to [18,19] for an introduction to CNNs and LSTM networks. The input consists of raw data being fed to six convolutional layers, where the features are learned and extracted. The choice of kernel sizes followed AlexNet [20], starting with a big kernel size (6×6) and then using smaller kernel sizes (3×3). In contrast, the number of filters increases for deeper layers. For performing the convolutional operations, zero padding is used. In the pooling layers the kernel sizes are 2×2 in the first and last layer and 3×3 in the second. Additionally, max-pooling is performed after every second layer, which is a common method to avoid overfitting and to reduce the computational effort. After the final convolutional layer, the resulting (flattened) feature vector then serves as input for two LSTM layers, two dense layers and one output layer, holding five output neurons. The LSTM layer is supposed to learn temporal dependencies within the sequences. All weights are initialized with the method presented by Xavier Glorot [21], and the Adam optimizer is used. The LSTM layer uses half the number of neurons as samples involved in one sequence. The first dense layer holds 1024 neurons and the second one 256. For better generalization, L2 regularization is used for the convolutional layer and a dropout of 0.2 is used for the dense and LSTM layers.

### 2.4. Training the Model

To guarantee training success, invariance in the test persons has to be ensured. Performing the training on one subject after another would not comply with that claim. Caused by the Adam optimizer algorithm [22], the learning rate would change constantly, and so, the network would be likely to overfit on the first person. Simply shuffling all the data is not a good option either, as this would neglect the temporal dependency. Taking this into consideration, we decided to cut the data into sequences of a certain length and shuffle those sequences. For the baseline model, the sequence length was 4 s (480 data points). The data were then fed to the network by using the sliding window approach. To do this, a window of fixed size was shifted along the time axis. The window shift was 1, leading to overlapping windows. As the convolutional network expects two-dimensional inputs, such as images, the windows containing *T* timestamps and N·C channels can be interpreted as such. Hereby *N* is the number of IMU sensors and *C* denotes the overall number of channels used. The baseline model uses 50 timestamps per window. The columns of this window are composed of 10 IMU sensors with 7 channels each, so input dimensions are 50×70. Raw data are fed to the network. No digital filters are used to smooth the signal.

Data from 11 subjects served as the training set within this study. The whole set of training, testing and validation procedures was performed according to the leave-one-subject-out validation method. This resulted in 10 training and 1 individual test subjects. This test subject was chosen randomly and is called the unseen subject in the following, as its data remains unseen to the model during training. The data of the remaining 10 subjects were also split into training (66%) and test sets (34%), whereby 10% of the training set was used for validation during the training. The tests were performed using both the test set and data of the unseen subject. The model converged after 10 epochs. Calculations were done at the THU on a workstation based on a NVIDIA GTX 1070Ti GPU, an AMD Ryzen 7 CPU with 3.7 GHz and 32 GB memory. On the software side, MATLAB (2018) and Python (version 3.6) have been used. The preprocessing routine, in terms of synchronization and interpolation, was implemented in MATLAB. Preparation and training of the model were done in Python. The final model was set up with Tensorflow (API r1.13); loading and managing the data were done with the pandas (version 0.23.4) and numpy (version 1.15.4) libraries. Matplotlib (version 3.0.1) was used for visualization of the data.

### 2.5. Hyperparameter Optimization

Finding the optimal set of hyperparameters can be very costly, due to the large amount of trainable hyperparameters. Fine tuning those parameters can result in a more stable and accurate model. Algorithms like grid search, which are often used, train the model for every joint specification of hyperparameter values in the Cartesian product of an individually chosen subset of the parameter space; the one with best validation results is chosen; see [23]. For reasons of limited computing power, the greedy approach is used in this paper, optimizing one parameter after the other, relying on experience and local best responses of the model. The drawback of this method is the risk that the globally best combination could be missed.

### 2.6. Feature Importance and Channel Order

Due to the fact that the network should be able to make online predictions, it is desirable to use as little data as possible. Since features are generated automatically by the convolutional layer, it is unclear which IMUs and channels have the most influence on the results—meaning, it is not only necessary to keep track of the data, but to also focus on the relevance of the individual channels. In order to obtain an insight into how the input representation affects the result, we analyzed, which channels are relevant and which order of channels leads to most auspicious results. The determination of most relevant data can be very costly. It would require the investigation of over three million combinations in order to determine which IMUs and channels provide the most important information to the network. Due to computational limitations, that is not an option. The strategy instead, is to analyze the activations in the convolutional layers, in order to get a feeling about the most relevant features when predicting and eliminating the other channels, again using a greedy approach. Here we observe whether the prediction accuracy can keep up further results.

**Figure 3:**
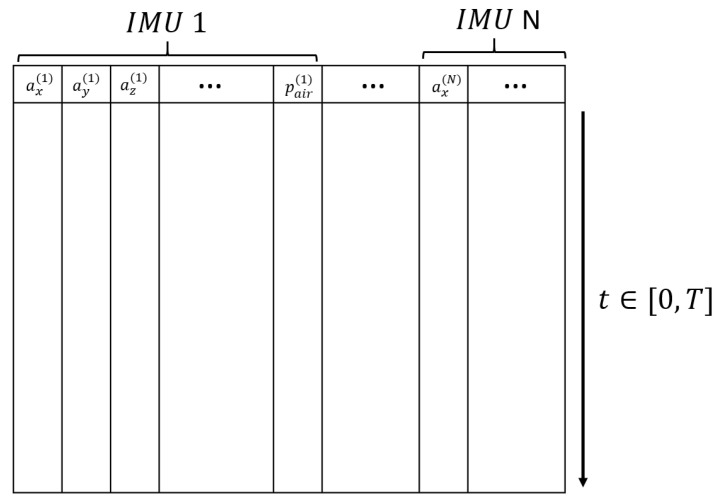
The figure shows how the channels are arranged in one window. All channels of an inertial measurement unit (IMU) are stringed together and are then merged with the channels of the other IMUs.

Yet another factor concerning the result of the model is about the order of the channels. Changing the order leads to different features, as the neighborhood in the convolutional operations is entirely different. We start by merging the channels of one IMU, as Figure 3 shows. Another surveyed configuration groups channels by their measurement ($a_{x}$, $a_{y}$, etc.). Both configurations will finally be compared in Section 3.3.

### 2.7. Network Certainty

As misclassifications in the medical field can have serious consequences, ensuring that misstatements are avoided is critical. As an indicator for good predictions, we define the output of the softmax function as certainty, while being well aware of the fact that this is not to be equated with the true correctness likelihood [24]:

(1) $$\begin{array}{c} Certainty{\left(z\right)}_{i}=\frac{exp(z_{i})}{\sum_{j=1}^{K}exp\left(z_{j}\right)},\mathrm{for}\;i=1,\ldots ,K\;\mathrm{und}\;z=\left(z_{1},\ldots ,z_{K}\right)\in R^{K} \end{array}$$

Post processing methods to avoid overconfidence, such as temperature scaling, are not used, as we do not need to know the exact probability to set thresholds. Since every prediction comes along with such a certainty, these will be analyzed for correct predictions and incorrect ones respectively. Thus, it can be considered whether it makes sense to allow predictions with maximum certainty only.

## 3. Results

**Figure 4:**
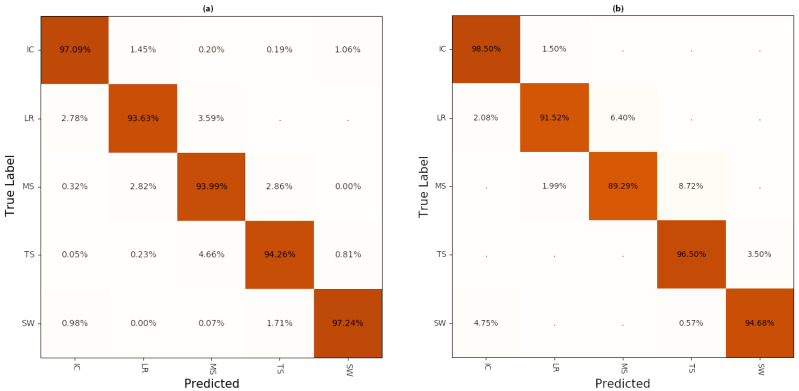
Confusion matrix of predictions applied to the test set (**a**), which consisted of 34% of the whole dataset and to one test person, who was not involved in the training process (**b**). On the main diagonal the correctly predicted labels can be seen.

After the training process finished and the model was validated and considered satisfactory, the test set was analyzed. While the validation set reached an accuracy of 97.24%, the test set reached 95.24%. In the confusion matrix in Figure 4, most misclassifications can be found on the first minor diagonal. It is also obvious that the swing phase and the initial contact have the best prediction rates.

Testing on an unseen subject results a slightly lower accuracy, but still, the same pattern can be seen (Figure 4b). It becomes evident that the prediction still reaches stable results, even though the network has not seen the data of the test person before. Additionally, the second and third minor diagonals have no entries, signifying that all misclassifications occur at the transition of two classes.

**Figure 5:**
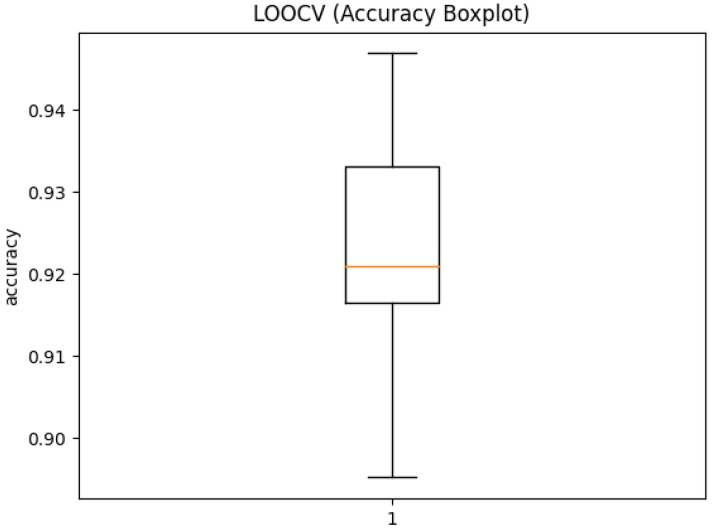
Box plot of the results of the LOOCV. For each model one subject was left out, and each model was tested for said subject. The median accuracy was 0.9209.

To prove that the approach is stable across all subjects, the leave-one-out cross-validation (LOOCV) method was used [25]. For each subject an individual model was trained, not involving that subject’s data. Table 1 shows the individual result for each subject, and in Figure 5 the corresponding box plot is depicted. Like this, we can show that the method generalizes reliably, when getting new data. The mean accuracy for all subjects is 0.9229 with a standard deviation of 0.01586.

### 3.1. Misclassification

**Figure 6:**
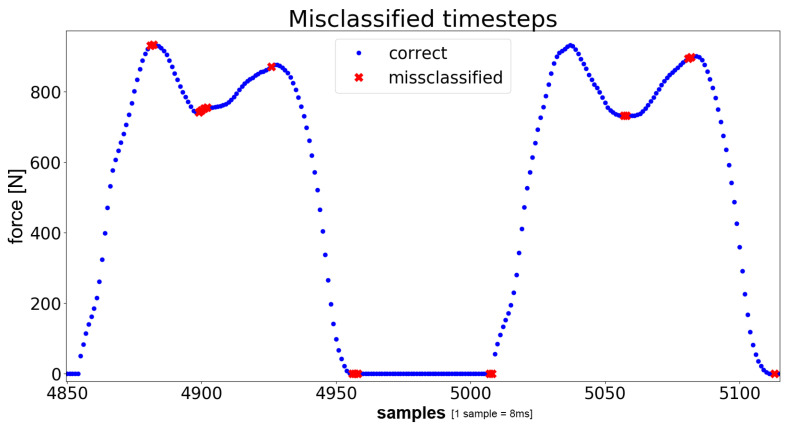
Correct (blue dots) and misclassified (red crosses) data samples in the label data. The y-axis indicates the total cumulated force [N].

In Figure 6, the values that were misclassified in the untrained dataset are exemplified. The blue dots show at which timestamps the correct label were predicted. To visualize the actual gait phase wherein the prediction fails, the corresponding pressure plate reaction was plotted. As can be seen, the errors are found at the local peaks (minima and maxima) of each step. Looking back at the label generation in Section 2.2, it is notable that most of the errors occur at the transitions between two classes, correlating with the results seen in the confusion matrices.

**Figure 7:**
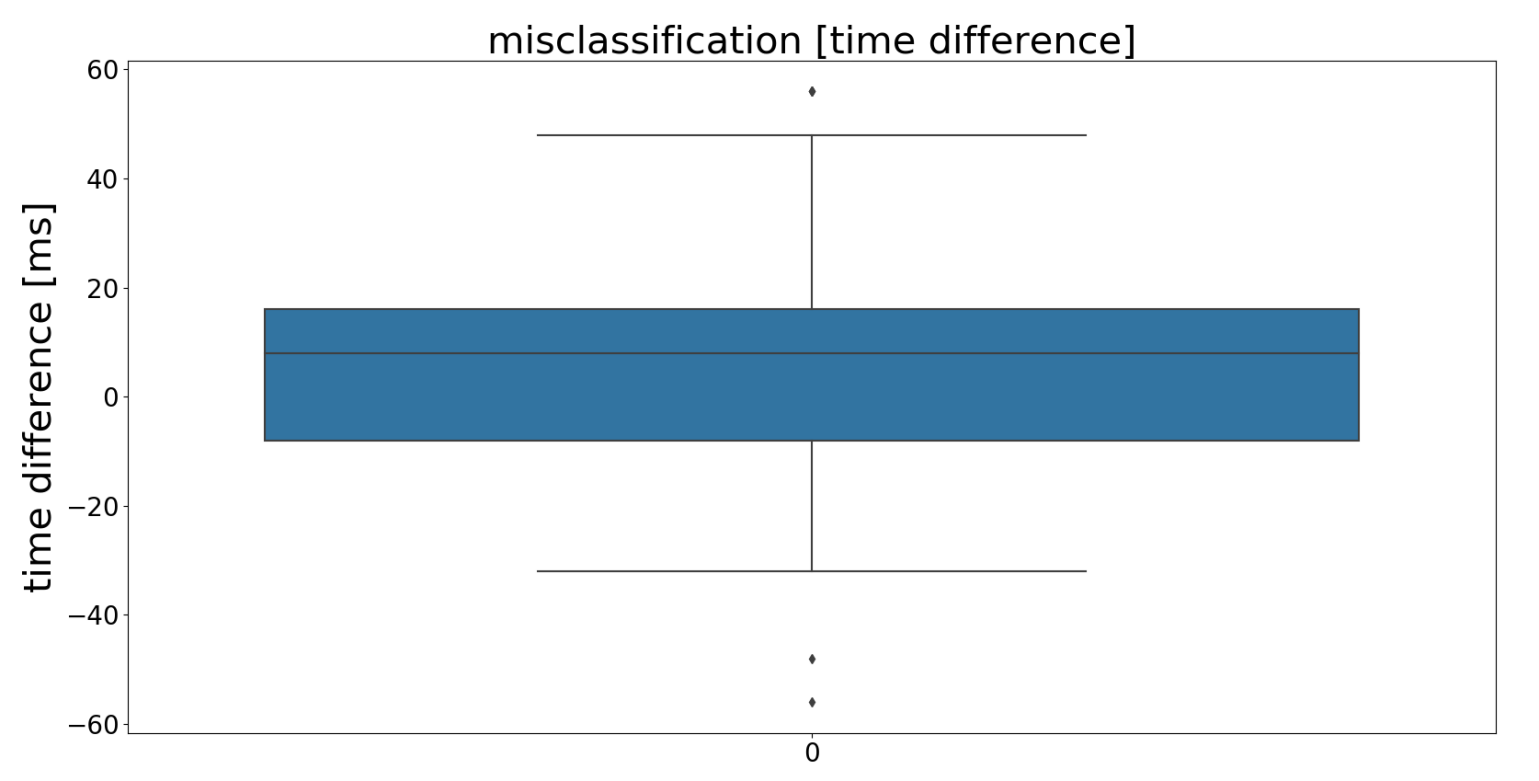
Time differences of mispredictions from the actual occurrences of events. The graph visualizes a box plot, representing the deviations in milliseconds (ms). The blue box shows the IQR (25th percentile (Q1) to the 75th percentile (Q3)), with the mean value being shown as a gray line inside the box. Besides that, minimum (Q1 −1.5· IQR), maximum (Q3 +1.5· IQR) and outliers are plotted.

To corroborate this hypothesis, the time differences between mispredictions and the actual occurrences of the events were measured and visualized in Figure 7. The results confirm the impression of the exemplary plot, visualized in Figure 6: The majority of false predictions missed the period by <16 ms (<2 time frames). The highest deviation was 56 ms (7 time frames). Thus, the assumption that transitions are most problematic was approved statistically.

To demonstrate the extent to which the accuracy is influenced by the temporal resolution of the IMU sensors, we define an acceptance criterion allowing false predictions, when missing the real class by 8, 16 and 24 milliseconds. For this experiment, data of an unseen subject were used. Table 2 shows how accurate the predictions are when the temporal uncertainty that originates in the process of label-generation is taken into consideration.

### 3.2. Hyperparameter Optimization

**Figure 8:**
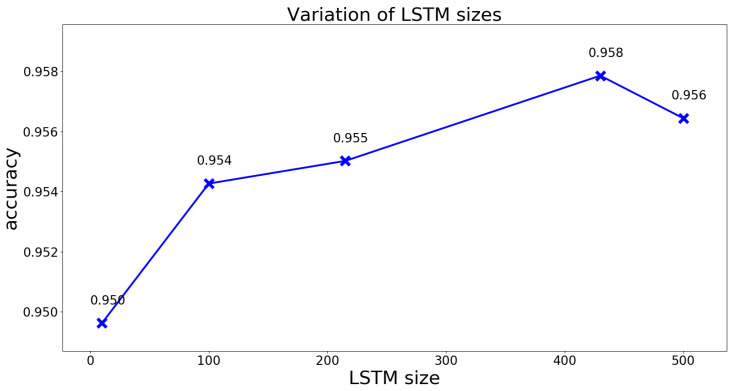
The influence of the LSTM layer size on the result. The test was done with the test dataset, containing unseen data of the test persons that have been involved in the training process.

Another important parameter influencing the result is the size of the LSTM layers. As the perfect number of hidden LSTM units is always dependent on the domain and input representation; five values were selected manually. The most obvious value is 480, which matches the number of windows in one sample. The idea would be to “remember” every value of the sequence before predicting the last one. Additionally, half the sequence length (=215), 100, 10 and a value greater than the sequence length (=500) were evaluated. Again, the model was tested on the unseen individual data, but also validated with the test set; see Figure 8.

As both figures confirm, the LSTM layer size, matching the sequence length, provides the best result and was therefore used in all further calculations.

### 3.3. Feature Importance and Channel Order

**Figure 9:**
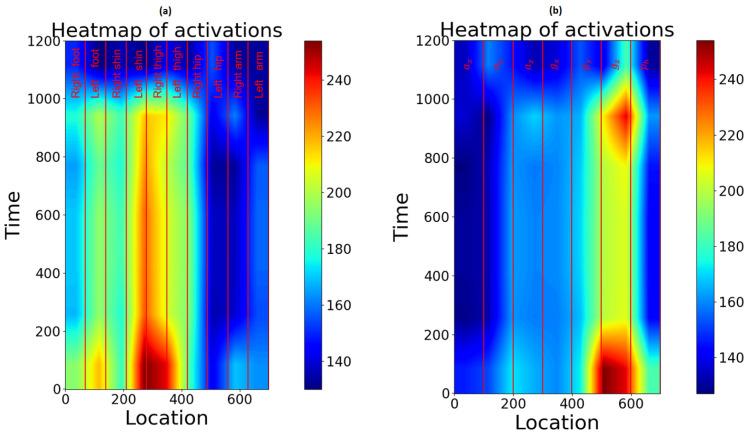
Activated regions in the last convolutional layer when predicting the gait phases. In (**a**) the channels are arranged device after device (IMU1, IMU2, etc.). In (**b**) the channels are grouped by the measurement type ($a_{x}$, $a_{y}$, $a_{z}$: acceleration; $g_{x}$, $g_{y}$, $g_{z}$: gyro data; p1: barometric pressure data).

In Figure 9, the activations of the last convolutional layer are shown in a heat-map. This way, spatial information about the most activated areas becomes visible. The activation was recorded, making predictions on unseen data. Note that absolute activations were averaged over all windows, normalized and resized: Due to the pooling operations, the last convolutional layer shrank the input to a matrix of only 7×9. For visualization purposes, this matrix was up-sampled to a 1200×700 Matrix.

The heat map shows that most activations can be measured in the shin or thigh area. Due to the low resolution, a precise assignment is not possible. For that reason, further tests have been conducted. The first approach was to take a look at each sensor’s location individually.

**Figure 10:**
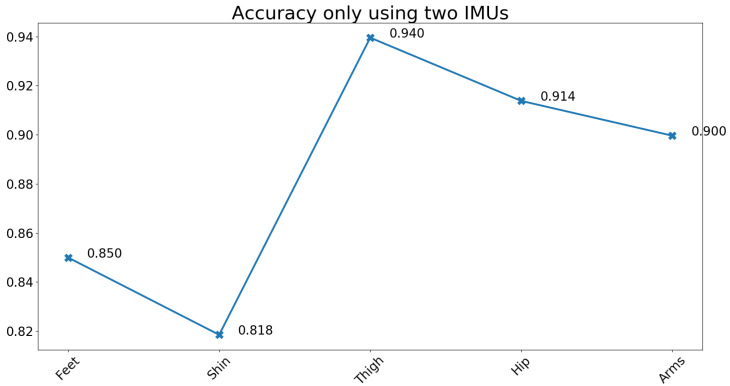
Accuracy of the classifier network reached by one pair of IMUs at different sensor locations, being trained individually. Training was again done with 10 subjects and tested on 1 unseen one.

The results of this experiment support the theory that features generated from the sensors located at the thighs provide the most beneficial information (see Figure 10). The overall result was enhanced, while prediction time and experimental expenditure decreased.

Using 2D-convolutional operations, the arrangement of the channels influences the result. When grouping the channels by their measurement ($a_{x}$, $a_{y}$, etc.), the accuracy decreases to 0.928. This suggests that spatial correlations have a greater influence on the result than those of the same relative direction.

Even though the accuracy decreases, it is noticeable that $g_{z}$ exhibits the highest activity.

For further reduction, the heat map, when using only the thighs, was analyzed. Again, the gyroscope data show higher activations. To find out if gyroscope data alone provides enough information for the classifier, we conducted training without acceleration data, only taking gyroscope data into account. This led to a significantly lower accuracy (0.894). For that reason, this approach was discarded.

### 3.4. Network Certainty

**Figure 11:**
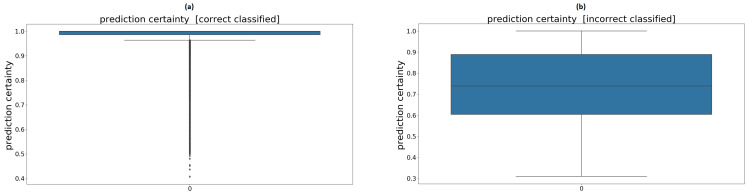
(**a**) Prediction certainty regarding correctly classified samples. (**b**) Prediction certainty regarding misclassified samples.

Figure 11a,b shows box plots of the prediction’s certainty when the prediction results are correct or incorrect.

As one would expect, the mean certainty of the correct classified samples was much higher than the that of the misclassified samples. Nevertheless, it should not be forgotten that the sample size of correct classified samples was larger than that of the incorrect ones. One idea is to declare all the samples under an intended certainty as unknown, to minimize the risk of drawing the wrong conclusions. The downside of this method is that correctly classified samples are also assigned to the unknown class.

Table 3 shows how the accuracy changes, if the certainty of the prediction is considered, and how many samples are assigned to the unknown class, if a threshold of 0.8 or 0.9 is chosen. These values relate to the evaluation of the untrained person’s data. With both thresholds, the accuracy rises, as over 60% of the originally misclassified samples are assigned to unknown. On the other hand, in numbers, more correctly classified samples are assigned to unknown than misclassified ones, leading to the fact that 8–14% of all the data are considered unidentified, whereas 6–10% of the correct ones are overruled. The use of this method has to be assessed as part of a risk analysis of the particular product and cannot be answered at this point.

## 4. Discussion

The ConvLSTM model achieved an accuracy higher than 92% on unseen data of new subjects. This result might not seem reliable enough for a medical application, but the analysis of where and why the errors occur shows the robustness of the model. Nearly all of the errors can be found at the transitions between classes. As we measured with a higher sampling rate than other papers we compared to above, difficulties at the transition become more visible. Measuring with 100 Hz only would most likely lead to higher accuracy values. To illustrate how small errors can lead to significant changes in accuracy, an average step is analyzed here. One step lasts about one second (120 measurements) and entails five transitions. Missing a transition on average by only one timestamp, would lead to $E=\frac{5}{120}\cdot 100=4.17\%$ misclassifications. Accordingly, to achieve an accuracy of 93% a transition is missed by an average of 1.12 time steps, where one time step is 8 ms. This lack of accuracy leads back to the label generation where the peaks of the pressure curve are calculated automatically to divide the signal into their gait phases. Since the pressure curves are not always perfectly smooth, it is not possible to divide the phases with an accuracy below one time frame. It is also possible that measurement errors in both IMU and treadmill data emerge. Unexpected movements like head-scratching or other arm movements could additionally influence the outcome, since the subjects were told to move as intuitively and naturally as possible. As our analysis shows, the accuracy rises when we accept a time uncertainty of one or more time frame. With three time frames, we already reached an overall accuracy above 99% testing on the unseen subject. This might also have been caused by the uncertainty in time synchronization explained in Section 2.2. With the available sampling rate provided by the IMU, it is hardly possible to gain a more detailed time resolution on the classification output.

The model was tested on 34% of the 10 training subjects, but the more significant test was done with data of one subject that had been left out for training, since these results are more critical, as can be seen when comparing the confusion matrices. Nevertheless, it is worth arguing whether the data of one subject are enough to evaluate the model’s ability to generalize. As the data generation and training is costly, switching training and test subjects to cross-validate the result has not been implemented.

It is also to be noted that all subjects were 35 years old or younger and healthy. There was no evidence that the model shows similar results with older people or people with abnormal gait characteristics.

## 5. Conclusions and Outlook

Throughout this paper, a deep neural network model based on a combination of CNN and LSTM was presented and comprehensively analyzed to predict the current gait phase while walking, using IMU sensors. Compared to previous approaches, labels are generated highly individually, and one step is separated into five phases. The model shows robust results, despite only being trained with ten subjects, reaching an accuracy of greater than 92% on unseen data, rising up to 99% if we accept a time deviation of up to three time steps. Although some issues remain unsolved within the scope of this work, the question of whether deep learning methods can build the foundation of a gait analysis system based on IMU sensors can be answered in the affirmative. Approaches to gain insights into the inner prediction process, with the aim of minimizing the number of parameters and the risk of mispredictions, have been presented and showed that optimization goes beyond simply testing a set of hyper-parameters. In future studies, more subjects should be used to have a broader test setup. In addition to this, a further study should be carried out examining the influences of older people and different gait patterns. Another promising attempt could be to use transfer learning, whereby the OPPORTUNITY dataset could be used to pre-train the convolutional network. With more computing power, it could also be possible to find a better hyperparameter set by using computationally costly methods, such as grid search.

All in all the results are very promising, and more accurate synchronization and labeling could lead to even more precise predictions.

## Figures and Tables

**Table 1:** Table showing the accuracy of each subject being left out during training.

Test Subject (Left Out)	Accuracy
subject 1	0.9218
subject 2	0.9469
subject 3	0.9204
subject 4	0.9428
subject 5	0.9451
subject 6	0.9209
subject 7	0.9161
subject 8	0.9020
subject 9	0.8952
subject 10	0.9168
subject 11	0.9234

**Table 2:** The accuracy of an unseen subject when temporal uncertainty at transitions is taken into consideration.

Accepted Offset to the True Class	0 Time Frames	1 Time Frame	2 Time Frames	3 Time Frames
	(0 ms)	(8 ms)	(16 ms)	(24 ms)
accuracy	0.9353	0.9422	0.9740	0.9907

**Table 3:** The change of accuracy when discarding samples under a chosen certainty is shown. Additionally, the table shows how many samples of the total dataset, the correct classified subset and the misclassified subset are assigned to “unknown” in consideration of the different certainties. The test set consists of the untrained subject not being retrained.

	Threshold 0.8	Threshold 0.9
accuracy (certainty > threshold)	0.9790	0.9880
unknown samples (amount)	2408	3866
correct → unknown (amount)	1544	2757
misclassified → unknown (amount)	864	1109
unknown samples (% of all samples)	0.0882	0.1416
correct → unknown (% of all correct samples)	0.05969	0.1066
misclassified → unknown (% of all false samples)	0.6017	0.7723

## Data Availability

Not applicable.
